# Take-home naloxone administered in emergency settings: feasibility of intervention implementation in a cluster randomized trial

**DOI:** 10.1186/s12873-024-01061-3

**Published:** 2024-08-29

**Authors:** Helen A. Snooks, Jenna K. Jones, Fiona B. Bell, Jonathon R. Benger, Sarah L. Black, Simon Dixon, Adrian Edwards, Helena Emery, Bridie A. Evans, Gordon W. Fuller, Steve Goodacre, Rebecca Hoskins, Jane Hughes, Ann John, Sasha Johnston, Matthew B. Jones, Chris R. Moore, Rakshita Parab, Richard Pilbery, Fiona C. Sampson, Alan Watkins

**Affiliations:** 1 Swansea University, Swansea, UK; 2 Yorkshire Ambulance Service, Wakefield, UK; 3 University of the West of England, Bristol, UK; 4 South Western Ambulance Services NHS Foundation Trust, Bristol, UK; 5 University of Sheffield, Sheffield, UK; 6 Cardiff University, Cardiff, UK; 7 Welsh Ambulance Services NHS Trust, Cardiff, UK; 8 Health Security Agency, London, UK

**Keywords:** Naloxone, Opioid-Related Disorders, Randomised controlled trial, Feasibility study, Emergency medical services

## Abstract

**Background:**

Opioids kill more people than any other class of drug. Naloxone is an opioid antagonist which can be distributed in kits for peer administration. We assessed the feasibility of implementing a Take-home Naloxone (THN) intervention in emergency settings, as part of designing a definitive randomised controlled trial (RCT).

**Methods:**

We undertook a clustered RCT on sites pairing UK Emergency Departments (ED) and ambulance services. At intervention sites, we recruited emergency healthcare practitioners to supply THN to patients presenting with opioid overdose or related condition, with recruitment across 2019–2021. We assessed feasibility of intervention implementation against four predetermined progression criteria covering site sign up and staff training; identification of eligible patients; issue of THN kits and Serious Adverse Events.

**Results:**

At two intervention sites, randomly selected from 4, 299/687 (43.5%) clinical staff were trained (ED1 = 107, AS1 = 121, ED2 = 25, AS2 = 46). Sixty THN kits were supplied to eligible patients (21.7%) (n: ED1 = 36, AS1 = 4, ED2 = 16, AS2 = 4). Across sites, kits were not issued to eligible patients on a further 164 occasions, with reasons reported including: staff forgot (*n* = 136), staff too busy (*n* = 15), and suspected intentional overdose (*n* = 3), no kit available (*n* = 2), already given by drugs nurse (*n* = 4), other (*n* = 4). Staff recorded 626 other patients as ineligible but considered for inclusion, with reasons listed as: patient admitted to hospital (*n* = 194), patient absconded (*n* = 161) already recruited (*n* = 64), uncooperative or abusive (*n* = 55), staff not trained (*n* = 43), reduced consciousness level (*n* = 41), lack of capacity (*n* = 35), patient in custody (*n* = 21), other (*n* = 12). No adverse events were reported.

**Conclusion:**

Staff and patient recruitment were low and varied widely by site. This feasibility study did not meet progression criteria; a fully powered RCT is not planned.

**Trial Registration:**

ISRCTN13232859 (Registered 16/02/2018).

## Background

In the UK and worldwide, fatal and non-fatal opioid poisoning is an increasing public health concern [1–3]. In England over half of drug deaths involve opioids such as heroin, methadone, fentanyl and morphine [4]. The number of deaths involving heroin and/or morphine doubled between 2012 and 2015 to the (then) highest on record [5].

People who misuse illicit or prescription opioids are at increased risk of non-fatal and fatal overdose, as well as long term morbidity, and they make high use of hospital Emergency Departments (ED) and ambulance services [4–10]. Emergency care contacts for drug-related morbidity have been shown to predict further overdoses [11, 12].

Naloxone is a safe and effective opioid antagonist routinely administered to people following opioid overdose by paramedics in the prehospital setting or ED staff [12]. Naloxone can also be supplied to lay people in the form of Take-Home Naloxone (THN).

Preliminary evidence from observational studies suggests that programmes providing kits including naloxone for administration by lay people in community settings are safe and effective [13–15]; however, concerns have been raised about the potential for over or underdosing [16]. Despite this lack of clarity over the safety and effectiveness of THN, national and international guidance (17–18) supporting its use has led to a proliferation of THN programmes in recent years in the UK and elsewhere, mostly distributed through specialist drugs services. (19–20) However, a significant proportion of people at risk of opioid overdose do not engage with these services [21].

We aimed to determine the feasibility of undertaking a fully powered RCT of THN in emergency settings by firstly testing whether implementing the intervention is feasible. We report methods and results of this study component in this paper. We also tested feasibility of methods to identify a population at high risk of fatal opioid overdose (to include in outcome comparisons); and carried out qualitative work to explore the acceptability of the intervention to stakeholders. These aspects are reported separately [22].

We intended, if all feasibility progression criteria were met, to propose a fully powered RCT to determine the safety, clinical and cost-effectiveness of the THN intervention.

### Objective

To assess the feasibility of implementing a THN intervention in a RCT clustered by sites comprising a paired Emergency Department (ED) and ambulance service; assessed against predefined progression criteria.

## Methods

The protocol for this parallel arm feasibility RCT clustered by site in 1:1 ratio has previously been published and we summarise methods here in accordance with CONSORT guidelines [23].

### Study setting

This study was clustered by sites formed by pairing ED and emergency ambulance service area. Sites were randomly allocated to intervention or control arms. Intervention sites are referred to as Site 1, comprising Emergency Department 1 (ED1) and Ambulance Service 1 (AS1); and Site 2: ED2 and AS2. Control sites 3 and 4 comprised ED3 with AS3, and ED4 with AS4.

### Inclusion and exclusion criteria

We included adult patients (aged 18 years or older) cared for by participating (TIME trained) ambulance paramedics or ED clinicians, for a presentation related to opioid use (e.g. opioid overdose or injuries due to opioid use), and assessed as having the capacity to consent to receive the THN kit and related training.

Patients were excluded if: known to have previously suffered an adverse reaction to naloxone; aggressive or exhibiting other challenging behaviours; already recruited; in police custody.

### Consent

We did not attempt to gain consent from patients to participate in the trial at the time of attendance for an opioid-related emergency. Although in some circumstances it may be ethical to gather consent to participation in research at the time of an emergency episode, we did not believe it would be possible to gain truly informed consent in the emergency setting, particularly when patients have just regained consciousness following an opioid overdose [24]. We did not try to gather consent retrospectively, as the population was deemed difficult to reach, and low contact rates could invalidate research findings. Patients were offered the option to opt-out from the study at all sites via patient information leaflets made available at ED waiting areas and supplied with THN kits. We also included this information on the Wales Centre for Primary and Emergency Care Research (PRIME) website (www.primecentre.wales).

### Clinical staff recruitment and training

Within intervention sites, ED nurses and doctors and ambulance service paramedics operating within the ED catchment area were invited to participate in the study. Volunteers were trained in delivering the THN intervention in accordance with the study protocol. Training, provided in a flexible manner to suit the working practices of individual departments and services, involved face-to-face group-based training, complemented by a ‘cascade’ approach whereby research support nurses and paramedics trained their peers on an ad hoc basis. Online resources produced by Martindale Pharma were available as refresher content for staff (http://www.prenoxadinjection.com/).

### Sample size

We aimed to include enough patients to test study design, methods and completeness of data. We expected to identify 200 records for individuals at high risk of overdose and thus eligible for the THN intervention in each site (100 via ED; 100 via the corresponding ambulance service) resulting in a total sample size at intervention sites of 400 participants. We did not carry out a power calculation to inform the sample size in this feasibility study as we were not aiming to determine effect sizes.

### Randomisation

A research team member (MJ) selected two sites as intervention and two as control sites at random from the set of all possible allocations, each contained within separate sealed opaque envelopes.

### Blinding

In this cluster RCT, it was not possible to blind participants or practitioners to allocation. The study statistician was not blinded when assessing the intervention implementation.

### Interventions

Usual care comprised supportive care and resuscitation as required plus naloxone administered by paramedics or ED clinicians in the case of overdose.

The THN intervention was offered to patients in addition to usual care and included a multi-dose THN kit (Prenoxad) containing 2 mg naloxone hydrochloride 1 mg/1 ml solution for intramuscular injection, and instructions on the correct administration of the naloxone dose. The kit contained simple instructions to back up training each participant received. Participants also received guidance on: basic life support; the importance of calling the emergency services; duration of effect; the safety of naloxone in terms of adverse events and overdose; and the legality of bystander administration of naloxone.

### Serious adverse events

We asked all participating sites to record any Serious Adverse Events that they became aware of, related to use of the THN kits. This included but was not restricted to deaths. We did not have any formal routine or systematic method of gathering this information, as the kits were for use by peers in the community.

### Outcomes

We assessed whether to proceed to a fully powered RCT using progression criteria, informed by a previous Cardiff-based feasibility study (CM, HS) [25], and confirmed by the independent Trial Steering Committee (TSC) in advance of data analysis.

The following criteria relate to the feasibility of implementing the THN intervention:

1. Sign up of four sites and ≥ 50% eligible staff to complete training in delivering the intervention at each intervention site;
2. Identification of ≥ 75% of people who presented to a participating ED or ambulance service with opioid overdose or related problem;
3. THN kits issued to ≥ 50% eligible patients at intervention sites;
4. Serious adverse event rate of no more than 10% difference between intervention and control sites.

### Changes to trial design

This trial was initially designed to allow THN to be given to friends and family of those at risk of opioid overdose, in line with drug service provisions. We were unable to proceed on this basis due to Patient Group Direction (required for supply of medications to non-clinicians) restrictions that kits had to be given to the person attended.

### Data analysis

We used straightforward descriptive statistics to address the four progression criteria relating to intervention implementation. No interim analyses were planned or performed.

## Results

**Fig. 1:**
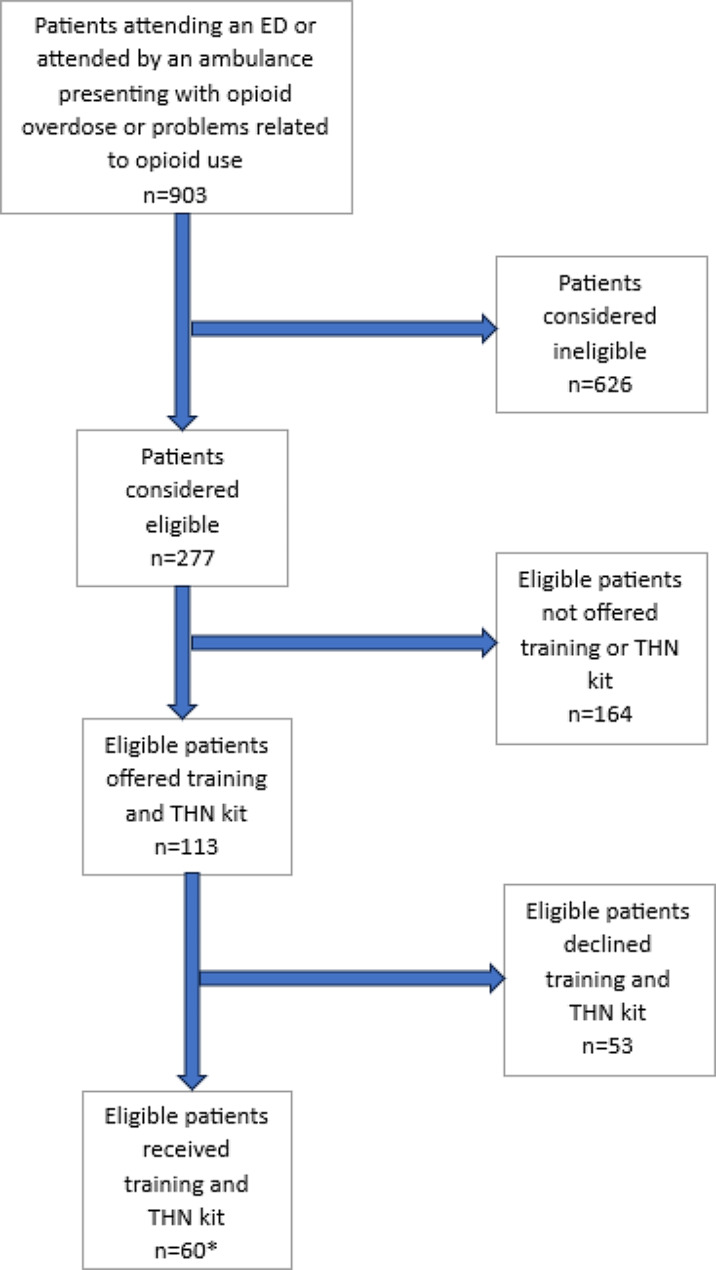
Participant recruitment *We omit further details on patients by site to mitigate the risk of inadvertently identifying individuals within small groups

Participant recruitment is shown in Fig. 1. Four sites participated. Across the two randomly allocated to the intervention arm, 299/687 (43.5%; n: ED1 = 107, AS1 = 121, ED2 = 25, AS2 = 46) eligible staff were trained to supply THN kits to eligible patients.

Sites opened for patient recruitment between May and October 2019. AS1 recruited for 61 weeks, AS2, ED1 and ED2 recruited for 52 weeks although these were non-consecutive due to closures at all sites during the Covid-19 pandemic.

In total, 277 patients were identified as eligible to receive the intervention during recruitment (Fig. 1). THN kits were supplied to sixty of these eligible patients (21.7%) during the recruitment period (n: ED1 = 36, AS1 = 4, ED2 = 16, AS2 = 4). In 16 cases (all in ED1), the patient initially agreed to the THN kit and accompanying training but ultimately did not receive the kit; 12 of whom were reported to already have a kit. In 37 cases, the patient declined the THN kit for reasons other than already having one (n: ED1 = 25, AS1 = 1, ED2 = 9, AS2 = 2).

Eligible patients were recorded as having not been offered THN kits on 164 occasions (n: ED1 = 159, AS1 = 2, ED2 = 0, AS2 = 3). Reasons reported for not offering eligible patients kits were: staff forgot (*n* = 136), staff too busy (*n* = 15), and suspected intentional overdose (*n* = 3), no kit available (*n* = 2), already given by drugs nurse (*n* = 4), other (*n* = 4).

Staff recorded 626 people as being considered for inclusion but found not to be eligible. Reasons for ineligibility were: patient admitted to hospital (*n* = 194), patient absconded (*n* = 161) already recruited (*n* = 64), uncooperative or abusive (*n* = 55), staff not trained (*n* = 43), reduced consciousness level (*n* = 41), lack of capacity (*n* = 35), patient in custody (*n* = 21), other (*n* = 12). No adverse events were reported.

We did not receive notice of any patient opt-outs.

### Assessment against progression criteria

Although some progression criteria were partially met - for example, in individual EDs or ambulance services – only the criterion related to patient safety was met across the trial, with no Serious Adverse Events reported (Table 1).

**Table 1:** Assessment of Progression Criteria related to intervention implementation

Progression Criterion	Relevant Result	Criterion Met?
1. Sign up of four sites, including ≥ 50% eligible staff to complete training in delivering the intervention at each intervention site	*Four sites participated in the trial; 299/687 (43.5%) eligible clinical staff were trained.*	No
2. Identification of ≥ 75% of people who have presented to ED or ambulance service with opioid overdose or related problem	*We were unable to identify all those who presented to ED or the ambulance service with opioid overdose or other related problem.*	No
3. THN kits issued to ≥ 50% eligible patients at intervention sites.	*THN kits were given to sixty of the 277 patients identified by trial staff as eligible (60/277 = 21.7%).*	No
4. Serious adverse event rate of no more than 10% difference in intervention sites to control sites	*No serious adverse events were reported.*	Yes

### Public and patient involvement and engagement (PPIE)

People affected by opioid overdose were directly involved throughout development of the research design [26–28], study conduct, and final report. They fully participated in the study – one as co-investigator and two as members of the Trial Management Group. We worked closely with voluntary, third sector and statutory groups supporting people affected by opioid overdose, including the Sheffield Addiction Research Recovery Panel (ShARRP). An independent Trial Steering Committee included two further public contributors. We supported all public contributors in line with the UK Standards for Public Involvement [26]. We offered honoraria and reimbursement of all expenses. We sought flexible routes to seek public contributions and communicate with individuals with relevant experience. We named a co-applicant [BAE] as PPIE Lead, who was supported by other research team members able to use their skills and geographic location for this aspect of our collaboration. We have reported our experiences in line with best practice [27].

## Discussion

### Key findings

Recruitment of clinical staff and distribution of THN kits was low, with considerable variation across participating EDs and ambulance services. Distribution of kits was particularly low in the prehospital setting.

### Limitations

The COVID-19 pandemic resulted in pauses in recruitment and increased pressures on the emergency services which may have affected staff recruitment and identification of eligible patients.

We relied on multiple informal routes for reporting of Serious Adverse Events related to use of the THN kits in the community. We acknowledge that this information may have been missing or incomplete.

### Implications for research, policy, practice

In this trial fewer THN kits were dispensed than expected, with the main reported reasons for considering the patient to be ineligible recorded as: admitted to hospital, absconded, abusive to staff; reduced capacity; and reduced consciousness level. Reasons given for not supplying THN to eligible patients included staff busy or forgot. A greater focus on relatives and friends may be prudent to the success of THN provision in emergency settings, although a recent European study about attitudes and likelihood of using THN kits reported that opioid users were significantly more likely to witness an overdose and use a THN kit compared to the family [29]. Research conducted in the United States reported that nearly half of the kits distributed by emergency services were given to family members with the patients themselves being the second largest group to receive the kit [30]. A study assessing the acceptance of naloxone nasal spray in the ED reported similar findings despite uptake being reported as low. Barriers included difficulties identifying the “right” patient; access to the kits; and lack of clarity as to when to offer the kit due to patients typically not waiting for formal discharge [31]. These findings are in line with other research which found the ED to be a suitable point for THN kit dispensing and training but reported ED staff did not have enough time for training and patient identification workflow, which could hinder the implementation of this intervention in the ED [32]. A more recent study assessing methods of increasing THN prescribing in the ED found that although barriers remain, improved, targeted staff training, and the use of work aids such as best-practice advisory tools, can increase the prescription of THN kits in the ED [33]. It may also be possible to identify patients for administration of a THN kit at the time of follow-up, rather than during the emergency episode.

## Conclusion

This feasibility study did not meet its predetermined progression criteria, and so funding for a fully powered trial will not be sought. However, we recognise that the emergency setting could be an important environment for identifying patients who may benefit from THN kit provision. Further research in this setting requires revisiting the intervention design in order to overcome issues faced during this feasibility trial.

## Data Availability

All data and study materials are available on request.

## References

[1] Snowdon J. Drug overdose death rates in different countries: who should be alarmed? Australasian Psychiatry. 2022;30(1):26–30. 35236130 10.1177/10398562221075192

[2] van Amsterdam J, van den Brink W, Pierce M. Explaining the differences in opioid overdose deaths between Scotland and England/Wales: implications for European opioid policies. Eur Addict Res. 2021;27(6):399–412. 33965949 10.1159/000516165 PMC8686715

[3] Pierce M, van Amsterdam J, Kalkman GA, et al. Is Europe facing an opioid crisis like the United States? An analysis of opioid use and related adverse effects in 19 European countries between 2010 and 2018. Eur Psychiatry. 2021;64(1):e47. 34165059 10.1192/j.eurpsy.2021.2219 PMC8316471

[4] Lewer D, Padmanathan P, ul Arfeen MQ et al. Healthcare use by people who use illicit opioids (HUPIO): development of a cohort based on electronic primary care records in England. Wellcome Open Res. 2020;5. 10.12688/wellcomeopenres.16431.1 PMC7901498 33659712

[5] Black C. Review of drugs: Phase one report. London: Gov.UK; 2020.

[6] O’Mara B. The effectiveness of changes to drug policy, regulation and legislation for reducing harms associated with opioids and supporting their medicinal use in Australia, Canada and the UK: a systematic review. Evid Base: J Evid Reviews key Policy Areas. 2020;2:79–110. 10.21307/eb-2020-004

[7] Alho H, Dematteis M, Lembo D, et al. Opioid-related deaths in Europe: strategies for a comprehensive approach to address a major public health concern. Int J Drug Policy. 2020;76:102616. 31855706 10.1016/j.drugpo.2019.102616

[8] Zibbell J, Howard J, Clarke SD, Ferrell A, Karon S. Non-fatal opioid overdose and associated health outcomes: final summary report. US Department of Health and Human Services; 2019. p. 33.

[9] Jiang R, Lee I, Lee TA, Pickard AS. The societal cost of heroin use disorder in the United States. PLoS ONE. 2017;12(5):e0177323. 28557994 10.1371/journal.pone.0177323 PMC5448739

[10] Warner-Smith M, Darke S, Day C. Morbidity associated with non‐fatal heroin overdose. Addiction. 2002;97(8):963–7. 12144598 10.1046/j.1360-0443.2002.00132.x

[11] Stoové MA, Dietze PM, Jolley D. Overdose deaths following previous non-fatal heroin overdose: record linkage of ambulance attendance and death registry data. Drug Alcohol Rev. 2009;28(4):347–52. 19594787 10.1111/j.1465-3362.2009.00057.x

[12] Ryan JM, Spronken I. Drug related deaths in the community: a preventive role for accident and emergency departments? Emerg Med J. 2000;17(4):272–3. 10.1136/emj.17.4.272 PMC1725416 10921816

[13] Yealy DM, Paris PM, Kaplan RM, Heller MB, Marini SE. The safety of prehospital naloxone administration by paramedics. Ann Emerg Med. 1990;19(8):902–5. 2372173 10.1016/S0196-0644(05)81566-5

[14] McDonald R, Strang J. Are take-home naloxone programmes effective? Systematic review utilizing application of the Bradford Hill criteria. Addiction. 2016;111(7):1177–87. 27028542 10.1111/add.13326 PMC5071734

[15] Walley AY, Xuan Z, Hackman HH, Quinn E, Doe-Simkins M, Sorensen-Alawad A et al. Opioid overdose rates and implementation of overdose education and nasal naloxone distribution in Massachusetts: interrupted time series analysis. BMJ. 2013;346. 10.1136/bmj.f174 PMC4688551 23372174

[16] Naloxone dosage for. opioid reversal: current evidence and clinical implications. Accessed 19 July 2024. https://www.ncbi.nlm.nih.gov/pmc/articles/PMC5753997/10.1177/2042098617744161 PMC5753997 29318006

[17] Organization WH. WHO-UNODC Stop Overdose Safely (SOS) initiative. 2020.

[18] ACMD. Consideration of naloxone. In: Drugs ACotMo, editor. 2012.

[19] Take-home naloxone EMCDDA. 2020. Accessed February 26, 2024. https://www.emcdda.europa.eu/publications/topic-overviews/take-home-naloxone_en#section6

[20] McDonald R, Campbell ND, Strang J. Twenty years of take-home naloxone for the prevention of overdose deaths from heroin and other opioids—conception and maturation. Drug Alcohol Depend. 2017;178:176–87. 28654870 10.1016/j.drugalcdep.2017.05.001

[21] Wales PH. Substance Misuse: Harm Reduction Database Wales (HRD): Drug related mortlaity Annual Report 2018-19 Accessed February 26, 2024 https://phw.nhs.wales/news/drug-deaths-at-their-highest-ever-levels-in-wales/harm-reduction-database-wales-drug-related-mortality-annual-report-2018-19/

[22] Sampson FC, Hughes J, Long J, Buykx P, Goodacre SW, Snooks H, Edwards A, Evans B, Jones J. Chris Moore, Sasha Johnston. Is a randomised controlled trial of take home naloxone distributed in emergency settings likely to be feasible and acceptable? Findings from a UK qualitative study exploring perspectives of people who use opioids and emergency services staff. BMC Emerg Med. 2024;24(1):75. 38679713 10.1186/s12873-024-00987-y PMC11057101

[23] Jones M, Bell F, Benger J, et al. Protocol for take-home naloxone in Multicentre Emergency (TIME) settings: feasibility study. Pilot Feasibility Stud. 2020;6:96. 10.1186/s40814-020-00626-w 32670598 10.1186/s40814-020-00626-w PMC7346647

[24] Good practice in research and Consent to research. General Medical Practice. 2020. Accesses February 26, 2024. https://www.gmc-uk.org/-/media/documents/Good_practice_in_research_and_consent_to_research.pdf_58834843.pdf

[25] Moore C, Lloyd G, Oretti R, Russell I, Snooks H. Paramedic-supplied ‘Take Home’Naloxone: protocol for cluster randomised feasibility study. BMJ open. 2014;4(3):e004712. 24650810 10.1136/bmjopen-2013-004712 PMC3963087

[26] UK Standards for Public Involvement. National Institute for Health Research. 2019. Accesses February 26, 2024. https://sites.google.com/nihr.ac.uk/pi-standards/home

[27] Staniszewska S, Brett J, Mockford C, et al. The GRIPP checklist: strengthening the quality of patient and public involvement reporting in research. Int J Technol Assess Health Care. 2011;27(4):391–9. 10.1017/S0266462311000481 22004782 10.1017/S0266462311000481

[28] Evans BA, Gallanders J, Griffiths L et al. Public involvement and engagement in primary and emergency care research: the story from PRIME Centre Wales. Int J Popul Data Sci 2020;5(3). 10.23889/ijpds.v5i3.1363 PMC7894248 33644413

[29] McDonald R, Eide D, Abel-Ollo K, Barnsdale L, Carter B, Clausen T, et al. A rapid assessment of take-home naloxone provision during COVID-19 in Europe. Int J Drug Policy. 2022;107:103787. 35849935 10.1016/j.drugpo.2022.103787 PMC9247228

[30] Langham S, Wright A, Kenworthy J, Grieve R, Dunlop WC. Cost-effectiveness of take-home naloxone for the prevention of overdose fatalities among heroin users in the United Kingdom. Value Health. 2018;21(4):407–15. 29680097 10.1016/j.jval.2017.07.014

[31] Bessen S, Metcalf SA, Saunders EC, et al. Barriers to naloxone use and acceptance among opioid users, first responders, and emergency department providers in New Hampshire, USA. Int J Drug Policy. 2019;74:144–51. 10.1016/j.drugpo.2019.09.008 31590090 10.1016/j.drugpo.2019.09.008 PMC7153573

[32] Lacroix L, Thurgur L, Orkin AM, Perry JJ, Stiell IG. Emergency physicians’ attitudes and perceived barriers to the implementation of take-home naloxone programs in Canadian emergency departments. Can J Emerg Med. 2018;20(1):46–52. 10.1017/cem.2017.390 28918769

[33] Funke M, Kaplan MC, Glover H, Schramm-Sapyta N, Muzyk A, Mando-Vandrick J, et al. Increasing naloxone prescribing in the emergency department through education and electronic medical record work-aids. Joint Comm J Qual Patient Saf. 2021;47(6):364–75. 10.1016/j.jcjq.2021.03.002 PMC8924938 33811002

